# Learning-dependent structural plasticity of intracortical and sensory connections to functional domains of the olfactory tubercle

**DOI:** 10.3389/fnins.2023.1247375

**Published:** 2023-08-23

**Authors:** Md Fazley Rabbi Sha, Yuriko Koga, Yoshihiro Murata, Mutsuo Taniguchi, Masahiro Yamaguchi

**Affiliations:** Department of Physiology, Kochi Medical School, Kochi University, Kochi, Japan

**Keywords:** olfactory tubercle, odor learning, plasticity, axonal boutons, olfactory bulb, piriform cortex

## Abstract

The olfactory tubercle (OT), which is a component of the olfactory cortex and ventral striatum, has functional domains that play a role in odor-guided motivated behaviors. Learning odor-guided attractive and aversive behavior activates the anteromedial (am) and lateral (l) domains of the OT, respectively. However, the mechanism driving learning-dependent activation of specific OT domains remains unknown. We hypothesized that the neuronal connectivity of OT domains is plastically altered through olfactory experience. To examine the plastic potential of synaptic connections to OT domains, we optogenetically stimulated intracortical inputs from the piriform cortex or sensory inputs from the olfactory bulb to the OT in mice in association with a food reward for attractive learning and electrical foot shock for aversive learning. For both intracortical and sensory connections, axon boutons that terminated in the OT domains were larger in the amOT than in the lOT for mice exhibiting attractive learning and larger in the lOT than in the amOT for mice exhibiting aversive learning. These results indicate that both intracortical and sensory connections to the OT domains have learning-dependent plastic potential, suggesting that this plasticity underlies learning-dependent activation of specific OT domains and the acquisition of appropriate motivated behaviors.

## Introduction

Odor cues induce a variety of motivated behaviors resulting in attraction to or aversion from the odor source. Recent advances are revealing the neural circuitry that links odor stimulation to motivated behaviors. Among various regions of the olfactory cortex, the olfactory tubercle (OT) is crucially involved in this neural circuitry (Heimer, 1978; Ikemoto, 2007; Wesson and Wilson, 2011). The OT is also called the tubular striatum as a component of the ventral striatum (Wesson, 2020). Its principal neurons, GABAergic medium spiny neurons, receive massive dopaminergic inputs from the ventral tegmental area (Millhouse and Heimer, 1984; Ikemoto, 2007). The OT plays a crucial role in odor-guided motivated behaviors (Fitzgerald et al., 2014; DiBenedictis et al., 2015; Wesson, 2020).

Although some odor signals elicit innate motivated behaviors (Ishii and Touhara, 2019; Mori and Sakano, 2021), many motivated olfactory behaviors are acquired through experience. The OT has been shown to be involved in the olfactory experience-dependent acquisition of behaviors (Gadziola et al., 2015; Zhang et al., 2017a; Gadziola et al., 2020; Millman and Murthy, 2020). We previously demonstrated that different domains of the OT become activated following attractive or aversive odor-based learning in mice (Murata et al., 2015). When mice were trained to associate an odor with a food reward and became attracted to the odor, the odor stimulus activated the anteromedial domain of the OT (amOT). In contrast, when mice were trained to associate the same odor with electrical foot shock and became aversive to the odor, the odor stimulus activated the lateral domain of the OT (lOT). The involvement of the medial OT in odor-attractive behaviors has been shown in several studies (DiBenedictis et al., 2015; Zhang et al., 2017a).

These findings indicate that the OT has highly plastic potential and that domain-specific activation induces corresponding motivated behaviors. However, neural mechanisms that enable the activation of specific OT domains in a learning-dependent manner remain unknown. The OT receives sensory input from the olfactory bulb (OB), as well as intracortical associational inputs from many brain areas, including the olfactory cortex, amygdala, prefrontal cortex, and ventral tegmental area (Haberly and Price, 1978; Berendse et al., 1992; de Olmos and Heimer, 1999; Zhang et al., 2017b; White et al., 2019; Cansler et al., 2023). Given that the activation of some brain regions depends on synaptic inputs from other brain regions, we hypothesized that synaptic inputs to OT domains are potentiated by olfactory learning and that this plastic change induces domain-specific activation of the OT.

As a first step to address this hypothesis, we examined the plastic potential of synaptic connections to OT domains by optogenetically activating neurons that project axons to the OT. We selected two synaptic connections, a sensory connection transferring sensory signals from the OB to the OT, and an intracortical connection from the piriform cortex (PC) to the OT. The PC is the widest subarea of the olfactory cortex and is the source of massive synaptic inputs to the OT (Haberly and Price, 1978; Narikiyo et al., 2014; White et al., 2019).

The relative plastic properties of sensory and intracortical connections in the olfactory cortex have been controversial (Kanter and Haberly, 1990; Bekkers and Suzuki, 2013). Some studies have shown less plasticity in the sensory connection to the PC (Poo and Isaacson, 2007; Johenning et al., 2009), whereas others have demonstrated its highly plastic potential (Cohen et al., 2015; Kumar et al., 2021). In this study, the optogenetic activation of OB- and PC-projection neurons was associated either with food reward or electrical foot shock, and the structural plasticity of the synaptic connections to OT domains was examined. Because synaptic bouton size is correlated with synaptic strength (Murthy et al., 2001; Sammons et al., 2018), we analyzed synaptic bouton size in projected neurons that terminated in the amOT and lOT.

## Materials and methods

### Animals

All experiments were conducted in accordance with the guidelines of the Physiological Society of Japan and were approved by the Kochi Medical School Animal Care and Use Committee. Neurotensin receptor 1-Cre recombinase transgenic mice [Nstr1-Cre mice; Tg(Ntsr1-cre)GN209Gsat, Mutant Mouse Regional Resource Center, University of California, Davis, CA, United States] were used to express channelrhodopsin (ChR2) in pyramidal cells in the PC. Protocadherin21-nCre recombinase transgenic mice [Pcdh21-nCre mice; Tg(Pcdh21-cre)BYoko, RIKEN BioResource Research Center, Ibaraki, Japan] (Nagai et al., 2005) were used to express ChR2 in mitral/tufted cells in the OB; they were mated with wild-type C57BL/6 mice (Japan SLC Inc., Shizuoka, Japan) and the male transgenic offspring were used for experiments. Genotyping of Ntsr1-Cre and Pcdh21-nCre mice was performed following standard polymerase chain reaction protocols using the following primers; Ntsr1-Cre, GACGGCACGCCCCCCTTA – CGGCAAACGGACAGAAGCATT; Pcdh21-nCre, ATGCCCAAGAAGAAGAGGAAGGTG – CGGATCCGCCGCATAACCAGTGA. After surgical intervention, the mice were individually housed in plastic cages (24 × 17 × 12 cm) with wood shavings at 26°C under a 12 h light/dark cycle, in which lights were switched on at 21:00 and off at 09:00.

### Stereotaxic surgery

Stereotaxic surgery was performed on 8–12-week-old mice. The mice were anesthetized with a mixture of three anesthetics (0.3 mg/kg medetomidine, 4 mg/kg midazolam, and 5 mg/kg butorphanol) and placed in a stereotaxic apparatus for virus injection and optic fiber implantation using a Cre-dependent adeno-associated virus expressing ChR2-mCherry fusion protein, AAV2-EF1a-DIO-hChR2-mCherry (5.1 × 10^12^ genome copies/mL, UNC Vector Core, Chapel Hill, NC, United States).

For ChR2 expression in PC pyramidal cells, Ntsr1-Cre mice were injected with the virus into the anterior PC (aPC) unilaterally (left) at the following coordinates: anterior from the bregma (A) 2.1 mm, lateral to the midline (L) 2.0 mm, deep to the brain surface (D) 3.6 and 4.0 mm; A 2.1 mm, L 2.2 mm, D 3.4 and 3.8 mm; A 2.1 mm, L 2.4 mm, D 3.2 and 3.6 mm; A 2.4 mm, L 2.0 mm, D 3.2 and 3.6 mm; and A 2.4 mm, L 2.2 mm, D 3.0 and 3.4 mm. We injected 0.1 μL/min of the virus for 4 min at each point. An optic fiber (DFC_480/500–0.63_4.0mm_MF_2.5FLT; Doric Lenses Inc., Sainte-Foy, QC, Canada) was implanted at A 2.25 mm, L 2.2 mm, D 3.00 mm, and fixed to the skull with dental acrylic.

To express ChR2 in OB mitral/tufted cells, Pcdh21-nCre mice were injected with the virus into the unilateral (left) OB by referring to the thick blood vessel between the OB and the frontal cortex. From the crossing point of the blood vessel and the midline, the injection coordinates were A 0.6 mm, L 0.6 mm, D 0.6 and 1.3 mm (posteromedial position of the OB); A 0.6 mm, L 1.1 mm, D 0.6 and 1.3 mm (posterolateral); A 1.0 mm, L 0.8 mm, D 0.55 and 1.3 mm (center); and A 1.4 mm, L 0.8 mm, D 0.55 and 1.3 mm (anterior). We injected 0.1 μL/min of virus for 1 min at each point. An optic fiber (DFC_480/500–0.50_1.0mm_MF_2.5FLT; Doric Lenses Inc.) was placed at the surface of the dorsal center of the OB and fixed to the skull with dental acrylic.

Injections were performed using a syringe pump (UltraMicroPump4; World Precision Instruments, Sarasota, FL, United States) connected to a Hamilton syringe (RN-1701; Hamilton Company, Reno, NV, United States) and a glass micropipette with a tip diameter of 80 μm. After each injection, the needle was left in place for an additional 30 s to allow the solution to spread evenly. After surgery, mice received an intraperitoneal injection of 0.3 mg/kg atipamezole to assist recovery from the anesthesia.

### Optogenetic stimulation

For optogenetic stimulation, the implanted optic fiber was connected to a blue light-emitting diode (465 nm; LEDC2-B/A_FC, Doric Lenses Inc.) and stimulated at ~10 mW. To stimulate aPC pyramidal cells, photostimulation was delivered for a duration of 20 ms at 20 Hz for 500 ms, which was repeated every 1 s. To stimulate OB mitral/tufted cells, it was delivered for a duration of 500 ms, which was repeated every 1 s. Photostimulation was triggered using a pulse generator (A310 Accupulser; World Precision Instruments).

### Photostimulation-food association training and behavioral assays

Training sessions were started at 2 weeks after virus injection and optic fiber implantation. During the training period, mice were food-restricted by delivering a limited amount of food pellets (2.7–3.3 g/day) to achieve 80–90% of their *ad libitum* feeding body weight. Water was available *ad libitum* throughout the experiment. Association learning and behavioral tests were conducted between 09:00 and 12:00, during the dark phase. Photostimulation–food reward association learning was conducted in a long plastic chamber (11 cm width × 20 cm height × 82 cm length). A mouse was confined in a left corner of the chamber using an opaque gate (~20 cm from the left wall) and powdered food was placed on a Petri dish in the opposite (right) corner. After 5 s of photostimulation, the gate was opened, and the stimulation continued until the mouse reached the powdered food and started eating. We conducted 30 trials per day, of which half included the treatment and food [(+) trial] and half included neither the treatment nor food [(−) trial]. The (+) and (−) trials were conducted randomly. In the first 5 s after the gate was opened, behavior was judged as correct if the mouse began to move to the opposite corner in a (+) trial or if it remained in the initial corner in a (−) trial. Training continued on consecutive days for a total of 9 days; the rate of correct behavior was calculated daily. Mice were judged to have learned appropriate behavior when the rate of correct behavior remained at >85% for 2 consecutive days. Control mice were photostimulated in the same chamber on the same schedule as the experimental mice, without food delivery. At day 10, the treatment was administered twice (15 times each, 5 s treatments, 40–60 s intervals) at an interval of 5 min to both trained and control mice, without food delivery. At 1 h after the initial administration of photostimulation, mice were perfusion-fixed for histological analysis.

### Photostimulation-foot shock association training and behavioral assays

Training sessions were started at 2 weeks after virus injection and optic fiber implantation. During the training period, food and water were provided *ad libitum*. Foot shock association learning and behavioral tests were conducted between 09:00 and 12:00, during the dark phase. Training was conducted in a long plastic foot shock chamber (11 cm width × 20 cm height × 57 cm length). Separate shock electrodes were installed on the right and left halves of the floor of the chamber, for separate activation (Choi et al., 2011). When mice remained in a corner (e.g., a left corner), photostimulation was delivered; 2 s later, electrical shock was delivered only to that side of the chamber (left side). Mice were able to escape the shock by moving to the opposite side (right side). The photostimulation continued until the mouse reached the opposite side. Mice gradually learned to avoid foot shock by moving quickly to the opposite side of the chamber following photostimulation. We conducted 10 trials per day, for 9 consecutive days. Mice were judged to have learned appropriate behavior when they moved to the opposite side of the chamber within ~2 s after the start of photostimulation more than 8 times among 10 trials. Control mice were photostimulated in the same chamber following the same schedule as the experimental mice. On day 10, the treatment was delivered without foot shock; this was repeated 10 times, at 1 min intervals, to both trained and control mice. One hour after the initial treatment, mice were perfusion-fixed for histological analysis.

### Histochemistry

Mice were deeply anesthetized via intraperitoneal injection of sodium pentobarbital (150 mg/kg), and then transcardially perfused with phosphate-buffered saline (PBS) followed by 4% paraformaldehyde (PFA) in 0.1 M phosphate buffer (PB). The brains were removed from the skull, immersed in 4% PFA overnight, and then transferred to 30% sucrose in 0.1 M PB. The brains were then embedded in optimal cutting temperature compound (Sakura Finetek, Tokyo, Japan), frozen at −80°C, and sliced into coronal sections at a thickness of 20 μm for OB and 30 μm for PC and OT using a cryotome. The sections were mounted on glass slides.

For immunohistochemical analysis, dried sections were rehydrated in PBS, blocked with Tris-buffered saline with 0.2% Triton-X (TBST) containing 10% normal goat serum, and incubated with primary antibodies in blocking buffer overnight at room temperature. The primary antibodies were mouse anti-Cre recombinase monoclonal antibody (1:200, MAB3120; Merck Millipore, Darmstadt, Germany), rabbit anti-c-fos polyclonal antibody (1:500; ab-190,289; Abcam, Cambridge, United Kingdom), mouse anti-mCherry monoclonal antibody (1:500; #632543; Takara Bio, Shiga, Japan), guinea pig anti-VGLUT1 polyclonal antibody (1:500; AB5905; Merck Millipore, Burlington, MA, United States), and rabbit anti-Homer 1b/c antibody (1:200; #160023; Synaptic Systems, Goettingen, Germany). The sections were washed with TBST and then incubated with appropriate fluorescent dye (Alexa)-conjugated secondary antibodies (1:300; Invitrogen, Waltham, MA, United States) for 1 h at room temperature. Then the sections were counterstained with 4′,6-diamidino-2-phenylindole (DAPI; 2 μg/mL) and mounted (Prolong Gold; Thermo Fisher Scientific, Waltham, MA, United States).

### Image acquisition and quantification

Image acquisition and quantification of mCherry(+) and c-fos(+) cells were conducted using a fluorescent microscope (DM600B; Leica, Wetzlar, Germany). For PC cell counting, serial coronal sections (30 μm thickness) were selected at a rate of one in every 10 and mCherry(+) pyramidal cells were counted. The dorsal edge of the lateral olfactory tract was used as a landmark to divide PC into medial and lateral subregions (Ekstrand et al., 2001). For OB cell counting, serial coronal sections (20 μm thickness) were selected at a rate of one in every 10 and mCherry(+) mitral and tufted cells were counted. For OT domain cell counting, serial coronal sections (30 μm thickness) of the OT were selected at a rate of one in every five and those containing amOT or lOT were analyzed. On average, we selected three sections for amOT and three sections for lOT for each mouse brain, and the density of c-fos(+) cells in amOT (layer II and Islands of Calleja) and lOT (layer II and cap compartments) was calculated.

Image acquisition and quantification of mCherry(+) axonal boutons in the OT domains were conducted using a confocal microscope (SP5; Leica). Serial coronal sections (30 μm thickness) of the OT were selected at a rate of one in every five and sections containing amOT or lOT were analyzed. On average, three sections for amOT and three sections for lOT were selected for each mouse brain. The size of each mCherry(+) bouton coexpressing VGLUT1 was measured from the image of one confocal XY plane (0.2 μm thickness) that exhibited the maximum size among serial Z images of the given bouton. Axonal bouton size (μm^2^) was measured using ImageJ (National Institutes of Health, Bethesda, MD, United States).

### Electrophysiological recordings

For PC recordings, Ntsr1-Cre mice were injected with AAV2-EF1a-DIO-hChR2-mCherry in the unilateral PC in the stereotaxic coordinates described above. After 2–3 weeks, mice were anesthetized with a mixture of three anesthetics. For local field potential (LFP) recording, a twisted tungsten electrode was placed in the PC (A 1.8 mm, L 2.2 mm, D 3.5 mm). For unit recording, a glass micropipette filled with 4 M sodium chloride (1.0–1.5 MΩ) was placed in the PC (A 2.2 mm, L 2.0 mm, D 4.0 mm), an optic fiber was inserted (A 2.0 mm, L 2.2 mm, D 3.0 mm), and photostimulation was delivered.

For OB recording, Pcdh21-nCre mice were injected with AAV2-EF1a-DIO-hChR2-mCherry in the unilateral OB in the stereotaxic coordinates described above. After 2–3 weeks, mice were anesthetized with a mixture of three anesthetics. For LFP recording, a twisted tungsten electrode was placed in the OB (A 0.6 mm, L 0.9 mm, D 1.0 mm; from the crossing point of the large vessel at the OB–cortex boundary and midline). For unit recording, a glass micropipette was placed in the OB (A 1.2 mm, L 0.8 mm, D 0.4–0.6 mm). An optic fiber was placed at the surface of the dorsal center of the OB, and photostimulation was delivered.

Electrical signals were amplified 1,000 times for LFP and 10,000 times for unit recording, and filtered to 0–300 Hz for LFP and 150 Hz–10 kHz for unit recording (Nihon Kohden, Tokyo, Japan). Data were stored in a computer via an analog/digital converter and analyzed using the Spike II software (Central Electrical Distributors, Rugeley, United Kingdom).

### Statistics

Statistical analyses were performed using GraphPad Prism software (version 7.04; La Jolla, CA, United States). One-way ANOVA, followed by Tukey’s multiple comparison test was used for comparisons among different olfactory areas and experimental conditions. Two-tailed Student’s *t*-test was conducted for comparisons between two groups. Statistical significance was set at *p* < 0.05. Graphs were constructed using GraphPad Prism software.

## Results

### ChR2 expression in projection neurons in the PC

**Figure 1:**
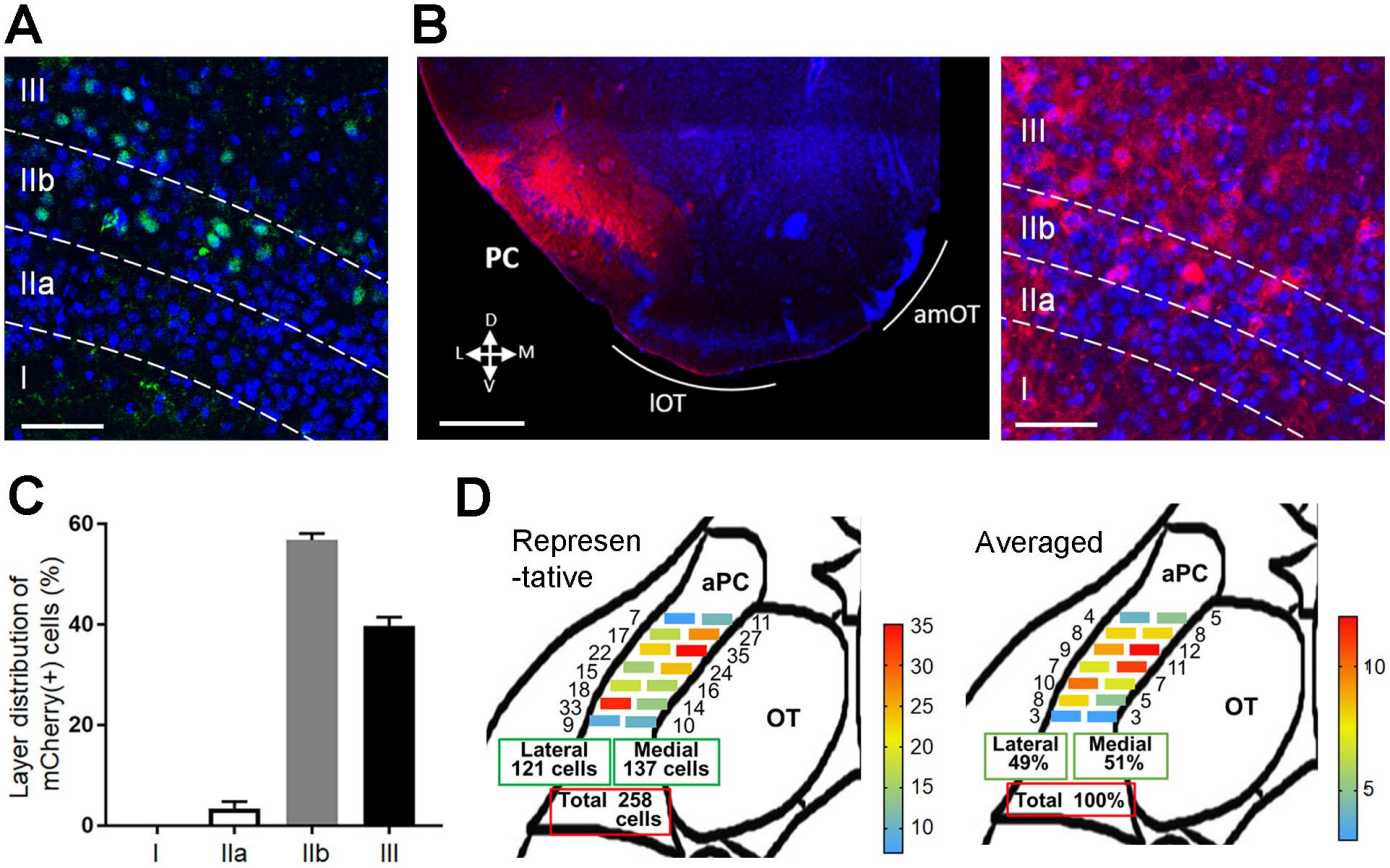
Expression of ChR2 in pyramidal cells of the PC in Ntsr1-Cre mice. **(A)** Cre recombinase expression in the PC of Ntsr1-Cre mice. Green, Cre recombinase; blue, DAPI. Layers I, IIa, IIb, and III are indicated. **(B)** ChR2-mCherry expression in the PC 2 weeks after the AAV injection (left panel). mCherry(+) cells were observed in layers IIb and III (right panel). Red, mCherry; blue, DAPI. D, dorsal; V, ventral; M, medial; L, lateral. **(C)** Percentage of mCherry(+) cells in the PC layers (average ± SD; *n* = 3 mice). **(D)** Distribution of mCherry(+) cells in the rostrocaudal and mediolateral axes of the PC. mCherry(+) cells were observed in the medial and lateral subregions of the anterior PC (aPC). In the representative data from one mouse (left schema), the numbers indicate the cell number observed in each subregion. In the averaged data (right schema, from 8 mice), the numbers indicate the percentage of cells distributing in each subregion. Scale bars; 50 μm [**(A,B)** right panel], 500 μm [**(B)** left panel].

We first examined the plastic potential of synaptic connections from the PC to OT domains using transgenic mice expressing Cre recombinase in pyramidal cells in the PC (Ntsr1-Cre mice). Cre recombinase was expressed in the deep portion of layers II (IIb) and III of the PC (Figure 1A). Adeno-associated virus expressing ChR2–mCherry fusion protein was injected into the unilateral anterior PC (aPC) of Ntsr1-Cre mice. After 2–3 weeks, the majority of mCherry(+) neurons were observed in layers IIb and III of the aPC (Figures 1B,C). Because axonal connection from the aPC to the OT is reported to be biased, such that medial aPC neurons project more to the lateral OT and lateral aPC neurons project more to the medial OT (Luskin and Price, 1983), we conducted a pilot study to achieve the unbiased distribution of mCherry(+) cells along the medial–lateral axis of the aPC, and optimized the stereotaxic coordinates of virus injection. Non-biased distribution of mCherry(+) cells occurred along the medial–lateral axis of the aPC in all mice subjected to behavioral and histological analysis; representative and summarized data are shown in Figure 1D.

**Figure 2:**
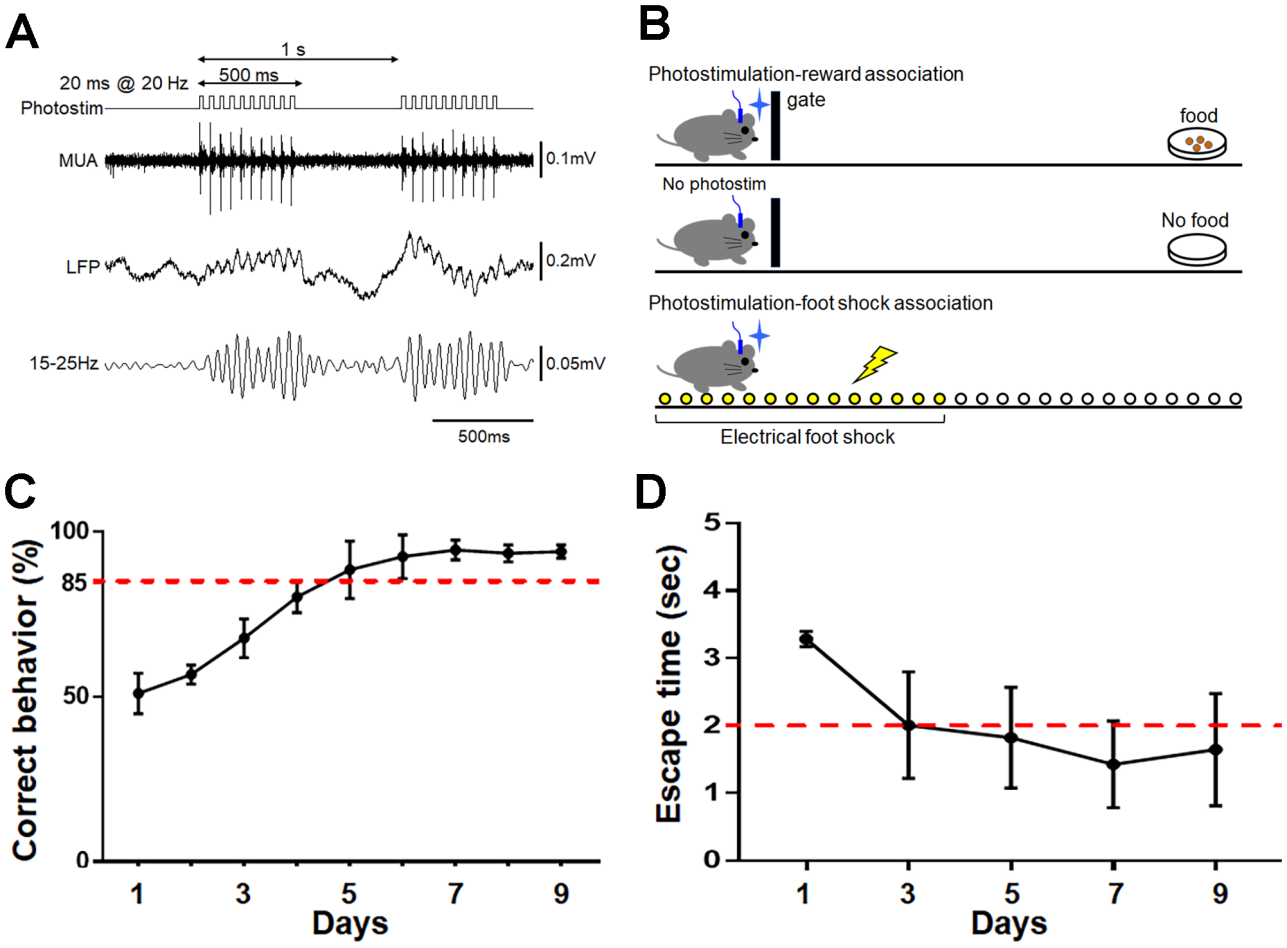
Photostimulation of aPC neurons and its association with reward or foot shock. **(A)** Protocol for photostimulation and evoked multi-unit activity (MUA) and local field potential (LFP) in the aPC. A 20 ms photostimulation was delivered at 20 Hz for 500 ms, and was repeated every 1 s. Oscillatory LFP faithful to photostimulation at 20 Hz is observed. **(B)** Schematic diagram of association learning. For reward association, the gate was opened following photostimulation to allow the mouse access to food at the opposite side of the chamber. Food was not provided when the gate was opened without photostimulation (top and middle schema). Photostimulation for foot shock association was delivered when the mouse remained in a corner, and an electrical shock was delivered only to that side of the chamber. Mice could escape the shock by moving to the opposite side of the chamber (bottom schema). **(C)** Acquisition of reward pursuit behavior. Rate of correct behavior is indicated. Mice learned appropriate go/no-go behavior during 9 days of training (>85% correct rate). Average ± SD, *n* = 4 mice. **(D)** Acquisition of shock avoidance behavior. Time taken to move to the opposite side of the chamber after the start of photostimulation (escape time) is indicated. Mice learned the appropriate behavior during 9 days of training (<2 s of escape time). Average ± SD, *n* = 4 mice.

To activate ChR2-mCherry-expressing neurons, photostimulation was conducted through the optic cannula implanted in the aPC. Photostimulation at 20 Hz (Choi et al., 2011) induced faithful oscillatory activity in the LFP and unit activity in the aPC (Figure 2A). The mice were trained to associate the treatment with either food reward or electrical foot shock (Figure 2B; see Materials and Methods). Mice trained for 9 consecutive days to perform reward pursuit behavior following photostimulation showed the correct go/no-go behavior at a rate of >85% (Figure 2C). Mice trained to perform shock avoidance behavior following such treatment showed rapid (within ~2 s) translocation to the shock-free side of the chamber (Figure 2D). These observations indicate that photostimulation of aPC neurons induced appropriate attractive or aversive behavior. Control mice received the same amount of stimulation per day on the same schedule as trained mice, without food reward or electrical foot shock; they showed no apparent attractive or aversive behaviors following it. On day 9, control mice that received photostimulation without food reward moved to the opposite side of the chamber (where the empty dish was placed) within 5 s in only 6.7 ± 5.4% of photostimulation (+) trials and 1.7 ± 3.3% of photostimulation (−) trials (average ± SD, *n* = 4 mice). Control mice that received photostimulation without foot shock did not move to the opposite side of the chamber even within 20 s in 90.0 ± 8.2% of trials (*n* = 4 mice).

### Activation of specific OT domains via photostimulation of aPC neurons following the association of photostimulation with reward or shock

On day 10, trained and control mice received photostimulation without reward or shock. It consistently elicited food searching behavior (moving to the place where powdered food was originally provided) in attraction-trained mice and aversive behavior (moving to the opposite side of the foot shock chamber) in aversion-trained mice. Attraction-trained mice moved toward the opposite side of the chamber very rapidly in 76.7 ± 2.7% of photostimulation (+) trials even though powdered food was not provided (*n* = 4 mice), while the control mice moved slowly to the other side of the chamber (apparently unintentionally) in 8.3 ± 6.9% of photostimulation (+) trials (*n* = 4 mice). Aversion-trained mice moved to the opposite side of the chamber very rapidly at 1.6 ± 0.8 s following the photostimulation although electrical shock was not delivered (*n* = 4 mice), while the control mice did not move even within 20 s in 75.0 ± 17.3% of trials (*n* = 4 mice).

**Figure 3:**
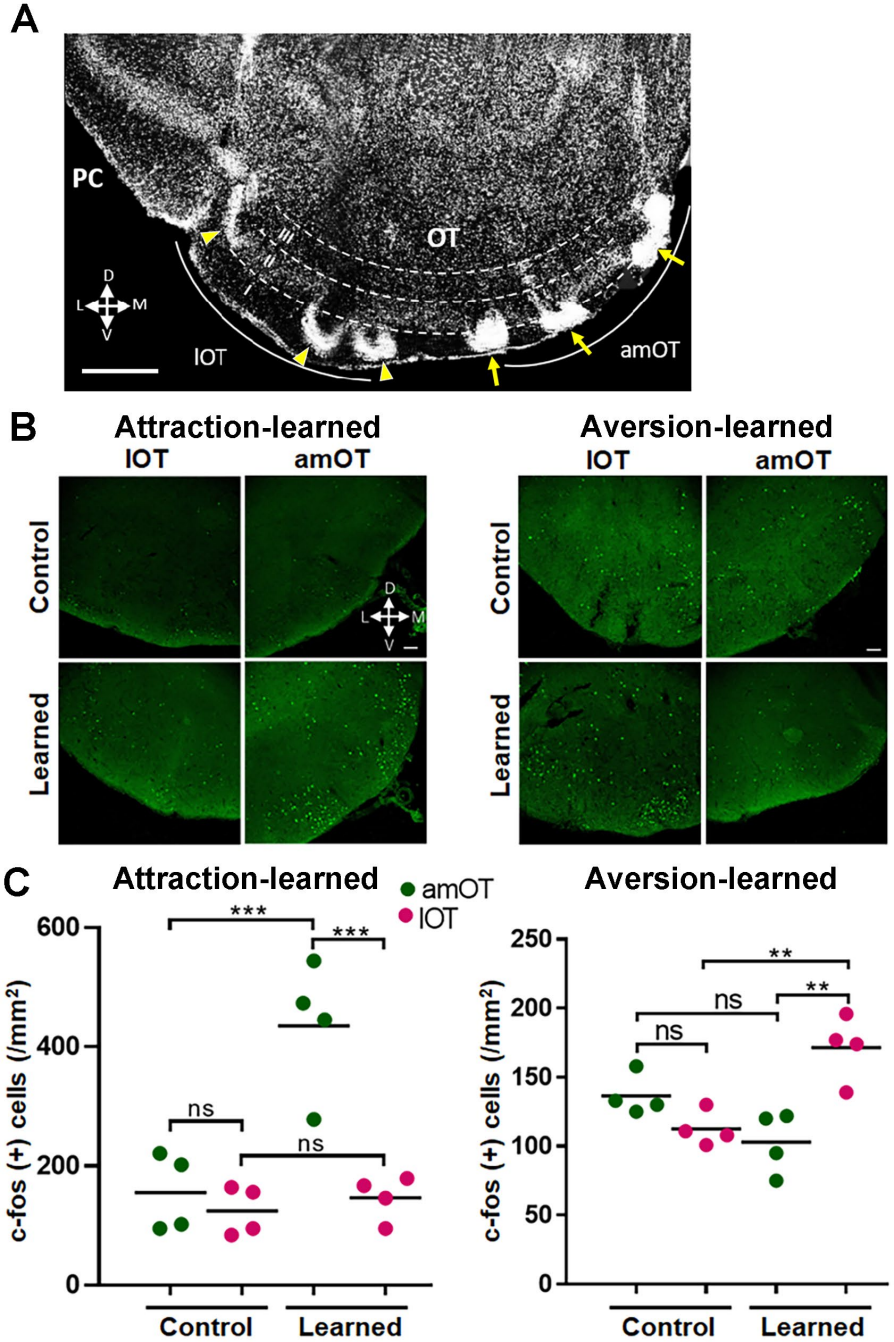
OT domain-specific activation by photostimulation of aPC neurons in association-learned mice. **(A)** Domain structure of the OT. A coronal section of the OT is indicated. Anteromedial (am) OT is surrounded by the superficial Islands of Calleja (arrows). Lateral (l) OT is surrounded by the cap compartments (arrowheads). **(B)** Distribution of c-fos(+) cells (green) in the amOT and lOT of the control and attraction-learned mice (left panels) as well as the control and aversion-learned mice (right panels). **(C)** Density of c-fos(+) cells in the amOT (green dots) and lOT (red dots) of the control and attraction-learned mice (left panel) as well as the control and aversion-learned mice (right panel). *n* = 4 mice per group, and lines indicate the averages. ns, not significant; **, *p* < 0.01; ***, *p* < 0.001 (one-way ANOVA with Tukey’s multiple comparison test). Scale bars; 500 μm **(A)**, 50 μm **(B)**.

The mice were perfusion-fixed at 1 h after the start of the treatment, and then were subjected to histological analysis. In the coronal sections of the OT, amOT was defined as the area surrounded by superficial Islands of Calleja, and lOT as the area surrounded by cap compartments (Figure 3A; Hosoya and Hirata, 1974; Fallon et al., 1978; Murata et al., 2015). In mice trained to perform reward pursuit behavior following the treatment, c-fos(+) cells were preferentially increased in the amOT (*F*_(3, 12)_ = 17.21, *p* < 0.0001; Figures 3B,C left panels; Supplementary Table S1 for multiple comparisons), whereas in mice trained to perform shock avoidance behavior following the treatment, c-fos(+) cells were preferentially increased in the lOT (*F*_(3, 12)_ = 10.4, *p* = 0.0012; Figures 3B,C right panels; Supplementary Table S1). These observations indicate that the association of aPC neuron photostimulation with either reward or shock induced amOT- or lOT-specific activation.

### Structural plasticity of axonal connections from the aPC to OT domains by the association of aPC neuron photostimulation with reward or shock

**Figure 4:**
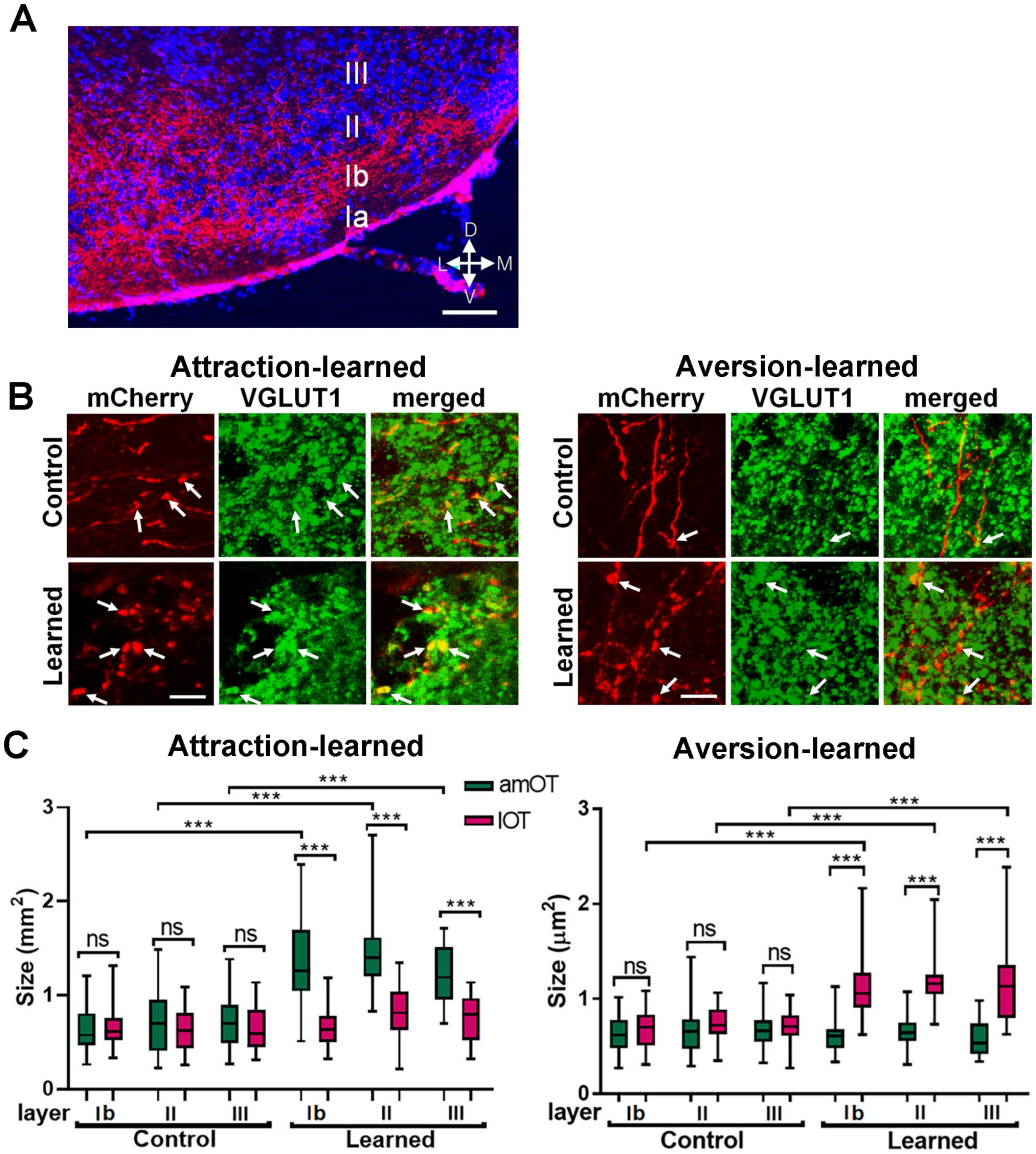
OT domain-specific size development of aPC neuron axonal boutons in association-learned mice. **(A)** Distribution of axons of mCherry(+) PC neurons (red) in layers Ib, II, III of the OT. Blue, DAPI. **(B)** mCherry(+) aPC neuron axonal boutons (red) coexpressing VGLUT1 (green) in the OT domains. Axonal boutons in amOT layer Ib for the control and attraction-learned mice (left panels) as well as in lOT layer III for the control and aversion-learned mice (right panels) are indicated. Arrows indicate mCherry(+) and VGLUT1(+) axonal boutons. **(C)** The size of the axonal boutons in layers Ib, II, and III of the amOT (green boxes) and lOT (red boxes) for the control and attraction-learned mice (left panel) as well as for the control and aversion-learned mice (right panel). Boxes indicate the 25th and 75th percentiles, whiskers indicate the minimum and maximum values, and lines inside the boxes indicate the median. Data were obtained from 4 mice per group. ns, not significant; ***, *p* < 0.001 (one-way ANOVA with Tukey’s multiple comparison test). Scale bars; 100 μm **(A)**, 5 μm **(B)**.

Next, we examined the possibility of structural plasticity in synaptic connections from the aPC to OT domains. Axons of mCherry(+) aPC neurons distributed in layers Ib (the deep portion of layer I), II, and III of the OT (Figure 4A). mCherry(+) axonal boutons in the amOT and lOT were identified by the coexpression of VGLUT1, a glutamatergic presynaptic vesicle protein (Takamori, 2006), under confocal microscopy (Figure 4B). The size of axonal boutons in each layer was compared between the amOT and lOT.

In control mice, axonal bouton size did not significantly differ between the amOT and lOT in each layer (Figure 4C). However, in mice trained to perform reward attraction behavior following the treatment, they became larger in the amOT than in the lOT in all layers (*F*_(11, 326)_ = 29.6, *p* < 0.001; Figure 4C left panel; Supplementary Table S2 for multiple comparisons). In mice trained to perform shock avoidance behavior following photostimulation, axonal boutons became larger in the lOT than in the amOT in all layers (*F*_(11, 445)_ = 34.2, *p* < 0.0001; Figure 4C right panel; Supplementary Table S2). The numbers of axonal boutons analyzed were 32, 28, 26, 37, 28, 27, 42, 28, 26, 25, 18 and 21 from left to right columns of Figure 4C for attraction learning and 39, 49, 39, 40, 29, 38, 42, 36, 33, 44, 32 and 41 for aversion learning, from 4 mice in each experimental condition. These results indicate that the association between aPC neuron photostimulation and either reward or shock induced amOT- or lOT-specific axonal bouton size development.

### Apposition of synaptic boutons of aPC neurons to postsynaptic structures in OT domains

**Figure 5:**
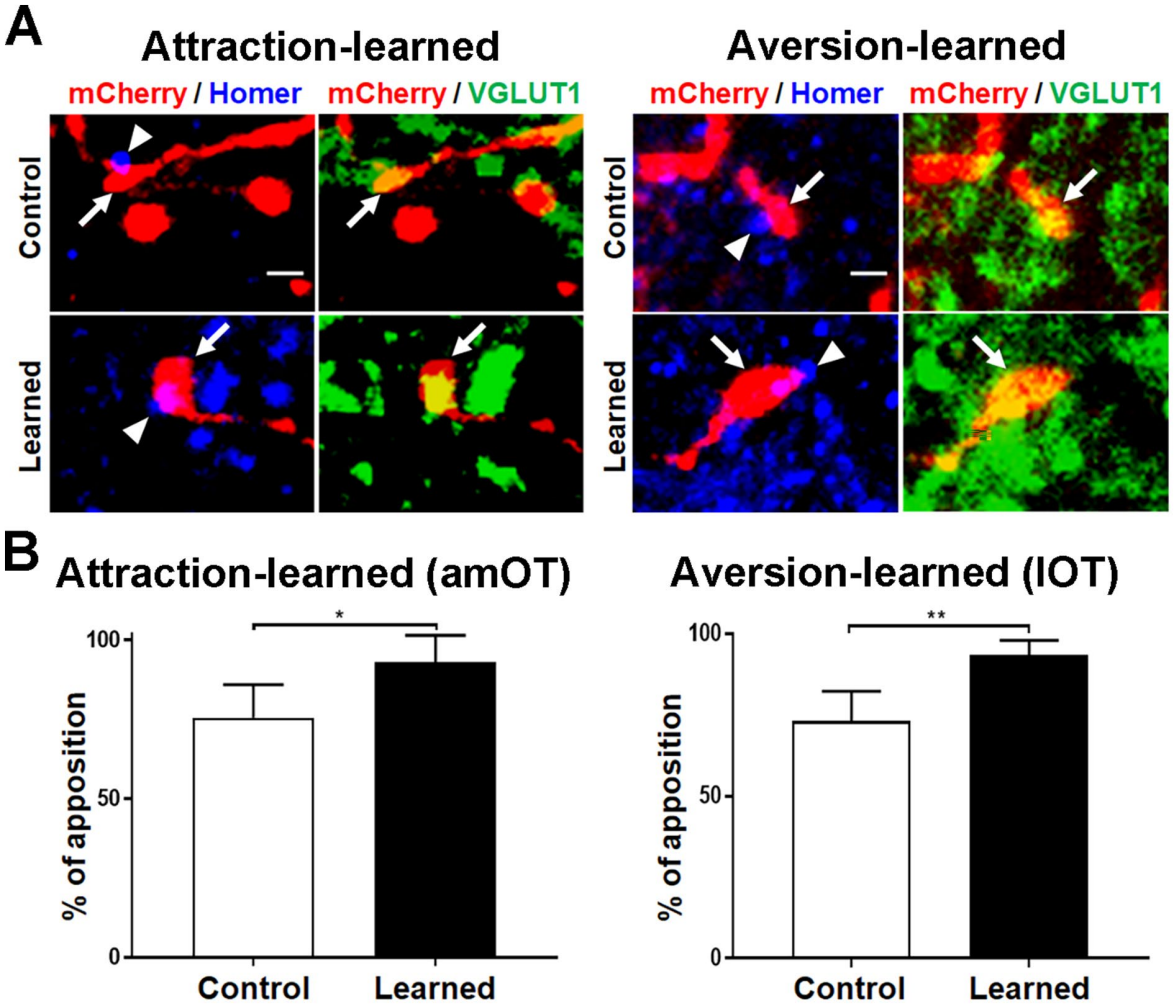
Apposition of aPC neuron axonal boutons to postsynaptic molecules. **(A)** Apposition of mCherry (red)/VGLUT1 (green) coexpressing aPC neuron axonal boutons (arrows) to Homer 1b/c puncta (blue) (arrowheads). Photographs of amOT layer Ib for the control and attraction-learned mice (left panels) and of lOT layer III for the control and aversion-learned mice (right panels) are indicated. Scale bar, 1 μm. **(B)** Percentage of apposition of axonal boutons to Homer 1b/c puncta in the amOT for the control and attraction-learned mice (left panel) as well as in the lOT for the control and aversion-learned mice (right panel). Average ± SD; *n* = 4 in each column, from rostrocaudally distinct 2 areas of layers Ib, II and III of 2 mice. *, *p* < 0.05; **, *p* < 0.01 (*t*-test).

To address whether mCherry(+) axonal boutons make synaptic contact with postsynaptic structures, we performed immunohistochemical analysis of Homer 1b/c, a post-synaptic scaffold protein for excitatory synapses (de Bartolomeis et al., 2022) in the amOT and lOT (Figure 5). Most mCherry(+) and VGLUT1(+) axonal boutons, including large ones in learned mice (Figure 5A lower panels), were apposed to the Homer 1b/c puncta. We quantified the distance between the mCherry(+) axonal boutons and the nearest Homer 1b/c puncta. From the distribution of the distances (Supplementary Figure S1) and previous reports (Ageta et al., 2001; Tao-Cheng et al., 2014), we set the criteria of <400 nm in the close apposition of the axonal boutons to the postsynaptic Homer 1b/c puncta. More than 70 and 90% of the axonal boutons were closely opposed to Homer 1b/c puncta in the amOT of control and attraction-learned mice, respectively (*p* = 0.040; Figure 5B left panel) as well as in the lOT of control and aversion-learned mice, respectively (*p* = 0.007; Figure 5B right panel). The total numbers of axonal boutons analyzed were 40 (control) and 46 (learned) for attraction learning and 41 (control) and 45 (learned) for aversion learning. The results suggest proper synapse formation of mCherry(+) axonal boutons.

### ChR2 Expression in mitral/tufted cells in the OB

**Figure 6:**
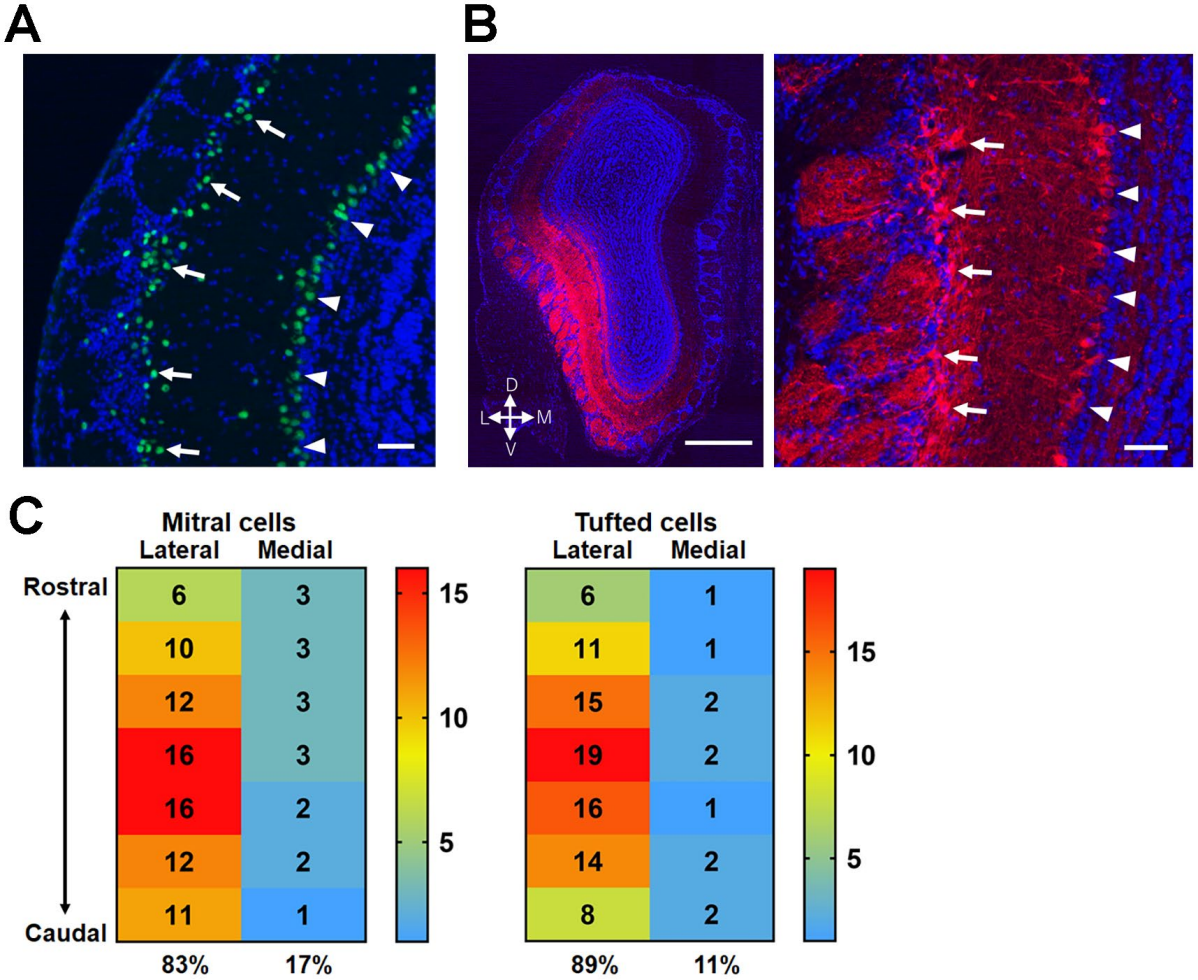
Expression of ChR2 in mitral/tufted cells in the OB of Pcdh21-nCre mice. **(A)** Cre recombinase expression in the OB of Pcdh21-nCre mice. Green, Cre recombinase; blue, DAPI. Mitral cells in the mitral cell layer (arrowheads) and tufted cells in the external plexiform layer (arrows) are indicted. **(B)** ChR2-mCherry expression in the mitral/tufted cells 2 weeks after the AAV injection. Red, mCherry; blue, DAPI. The majority of mCherry(+) cells are observed in the lateral wall of the OB (left panel). Magnified view indicates mCherry expression in mitral cells (arrowheads) and tufted cells (arrows) (right panel). **(C)** Distribution of mCherry(+) mitral cells (left panel) and tufted cells (right panel) in the rostrocaudal and mediolateral axes of the OB. Majority of mCherry(+) mitral/tufted cells were seen in the lateral half of the OB. The numbers indicate the percentages of cells distributed in each subregion (averaged from 6 mice). The indicated subregions span ~1.5 mm of central area along the rostrocaudal axis of the OB. Scale bars; 50 μm [**(A,B)** right panel], 500 μm [**(B)** left panel].

Next, we examined the plastic potential of synaptic connections from the OB to OT domains using transgenic mice expressing Cre recombinase in mitral/tufted cells in the OB (Pcdh21-nCre mice) (Figure 6A). Adeno-associated virus expressing ChR2–mCherry fusion protein was injected into the unilateral OB of Pcdh21-nCre mice. After 2–3 weeks, mCherry(+) mitral/tufted cells were observed in the OB. In our stereotaxic virus injection, mCherry(+) mitral/tufted cells were mainly distributed in the lateral wall of the OB (Figures 6B,C). The overall ratio of labeled mitral cell and tufted cell numbers was 0.75 ± 0.05 versus 0.25 ± 0.05 (arbitrary unit; average ± SD, 6 mice). The distribution of mCherry(+) cells in the lateral OB was confirmed in all mice subjected to behavioral and histological analyses.

**Figure 7:**
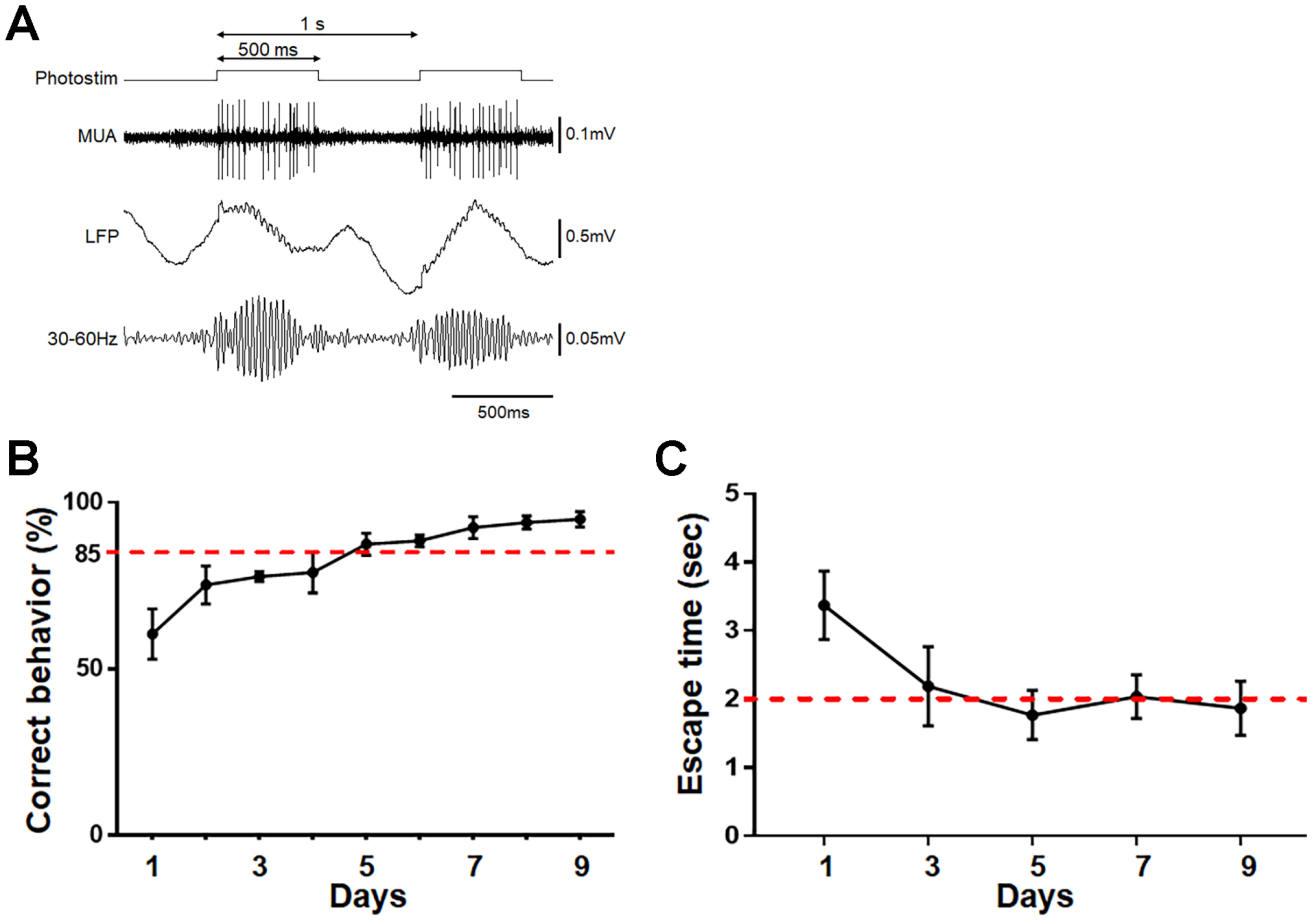
Photostimulation of OB neurons and its association with reward or foot shock. **(A)** Protocol for photostimulation, evoked multi-unit activity (MUA), and local field potential (LFP) in the OB. A 500 ms photostimulation was delivered every 1 s. Oscillatory LFP at ~40 Hz was observed. **(B)** Acquisition of reward pursuit behavior. Rate of correct behavior is indicated. Mice learned the appropriate go/no-go behavior during 9 days of training (>85% correct rate). Average ± SD, *n* = 4 mice. **(C)** Acquisition of shock avoidance behavior. Time taken to move to the opposite side of the chamber after the start of photostimulation (escape time) is indicated. Mice learned the appropriate behavior during 9 days of training (<2 s of escape time). Average ± SD, *n* = 4 mice.

Photostimulation of ChR2-mCherry-expressing mitral/tufted cells was conducted through the optic cannula implanted at the dorsal surface of the OB. Continual photostimulation for several hundred milliseconds (Arenkiel et al., 2007) induced oscillatory activity at beta – low gamma range in the deep OB layer (granule cell layer) and unit activity in the mitral cell layer (Figure 7A; Frederick et al., 2016). The mice were trained to associate the treatment with either a food reward or electrical foot shock. During 9 consecutive days of training, mice trained to associate the treatment with a food reward exhibited reward-pursuing behavior following the treatment, with a correct behavior rate of >85% (Figure 7B). Mice trained to associate it with electrical foot shock exhibited shock-avoiding behavior following the treatment, with rapid (< 2 s) translocation to the shock-free side of the chamber (Figure 7C). These observations indicate that photostimulation of mitral/tufted cells in the OB induced appropriate attractive or aversive behavior. Control mice received the same amount of stimulation on the same schedule as trained mice, without food reward or electrical foot shock, and showed no apparent attractive or aversive behaviors following it. On day 9, control mice that received photostimulation without food reward moved to the opposite side of the chamber within 5 s in only 10.0 ± 6.7% of photostimulation (+) trials and 3.3 ± 3.8% of photostimulation (−) trials (*n* = 4 mice). Control mice that received photostimulation without foot shock did not move to the opposite side of the chamber even within 20 s in 92.5 ± 5.0% of trials (*n* = 4 mice).

### Activation of specific OT domains via photostimulation of OB mitral/tufted cells following reward/shock association training

On day 10, both trained and control mice received photostimulation without reward or shock. It consistently elicited food searching behavior in attraction-trained mice and aversive behavior in aversion-trained mice. Attraction-trained mice moved toward the opposite side of the chamber very rapidly in 85.8 ± 4.2% of photostimulation (+) trials even though powdered food was not provided (*n* = 4 mice), while the control mice moved slowly to the other side of the chamber (apparently unintentionally) in 18.3 ± 2.7% of photostimulation (+) trials (*n* = 4 mice). Aversion-trained mice moved to the opposite side of the chamber very rapidly at 1.9 ± 0.2 s following photostimulation although no electrical shock was delivered (*n* = 4 mice), while the control mice did not move even within 20 s in 72.5 ± 12.6% of trials (*n* = 4 mice).

**Figure 8:**
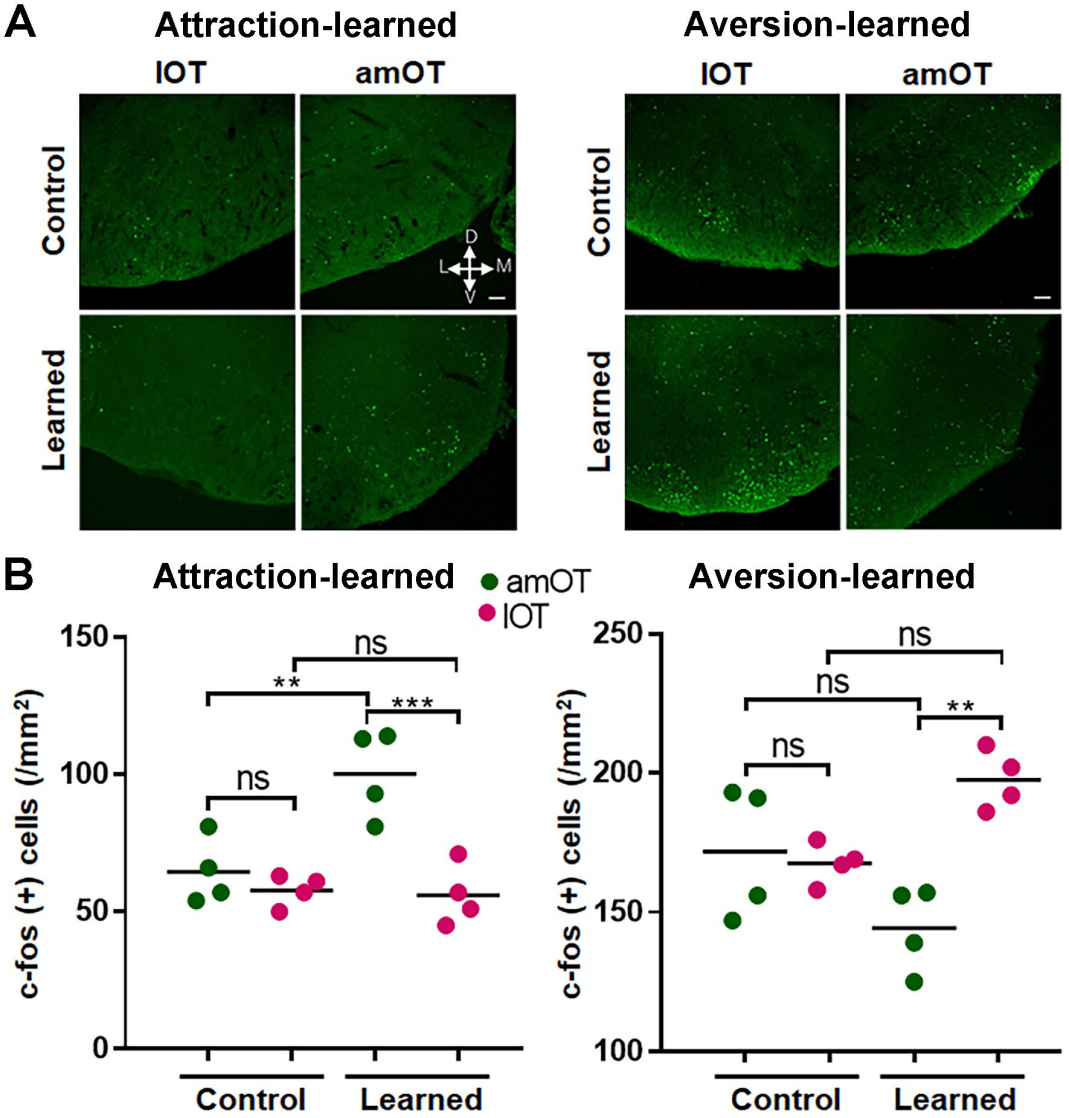
OT domain-specific activation by photostimulation of OB neurons in association-learned mice. **(A)** Distribution of c-fos(+) cells (green) in the amOT and lOT of the control and attraction-learned mice (left panels) as well as the control and aversion-learned mice (right panels). Scale bar, 50 μm. **(B)** Density of c-fos(+) cells in the amOT (green dots) and lOT (red dots) of the control and attraction-learned mice (left panel) as well as the control and aversion-learned mice (right panel). *n* = 4 mice in each group, and lines indicate the average. ns, not significant; **, *p* < 0.01; ***, *p* < 0.001 (one-way ANOVA with Tukey’s multiple comparison test).

The mice were perfusion-fixed 1 h after the start of the treatment and subjected to histological analysis. In mice trained to perform reward-pursuing behavior, c-fos(+) cells were preferentially increased in the amOT (*F*_(3, 12)_ = 12.25, *p* = 0.0006; Figures 8A,B left panels; Supplementary Table S3 for multiple comparisons), whereas in mice trained to perform shock avoidance behavior, c-fos(+) cells were preferentially increased in the lOT (*F*_(3, 12)_ = 7.92, *p* = 0.0035; Figures 8A,B right panels; Supplementary Table S3). These observations indicate that the association between OB neuron photostimulation and either reward or shock induced amOT- or lOT-specific activation.

### Structural plasticity of axonal connections from the OB to OT domains following reward/shock association training

**Figure 9:**
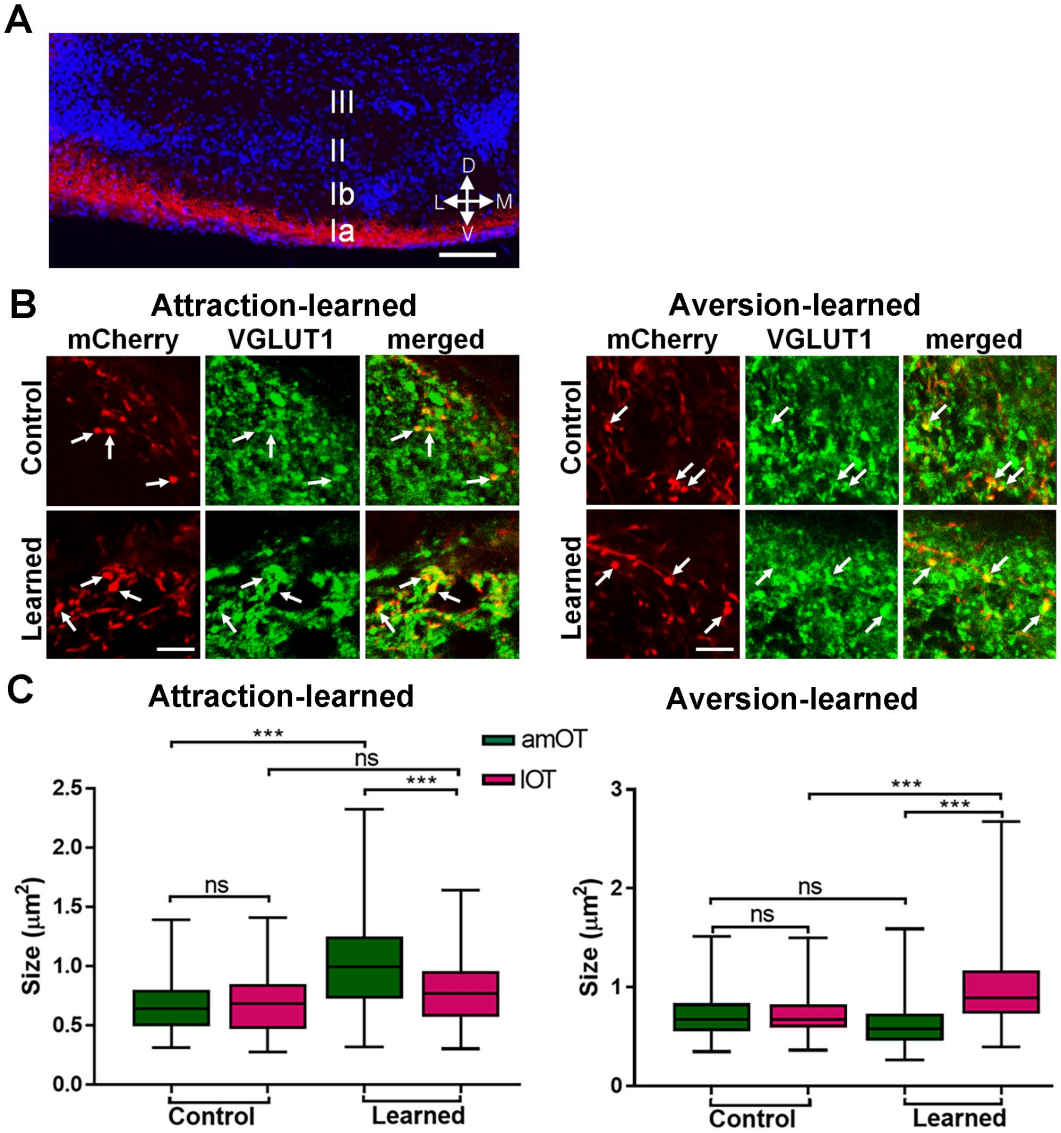
OT domain-specific size development of OB neuron axonal boutons in association-learned mice. **(A)** Distribution of axons of mCherry(+) mitral/tufted cells (red) in the layer Ia of the OT. Blue, DAPI. **(B)** mCherry(+) OB neuron axonal boutons (red) coexpressing VGLUT1 (green) in the OT domains. Axonal boutons in layer Ia of the amOT for the control and attraction-learned mice (left panels) and in layer Ia of the lOT for the control and aversion-learned mice (right panels) are indicated. Arrows indicate mCherry(+) and VGLUT1(+) axonal boutons. **(C)** The size of the axonal boutons in layer Ia of the amOT (green boxes) and lOT (red boxes) for the control and attraction-learned mice (left panel) as well as for the control and aversion-learned mice (right panel). Boxes indicate the 25th and 75th percentiles, whiskers indicate the minimum and maximum values, and lines inside the boxes indicate the medians. Data were obtained from 4 mice per group. ns, not significant; ***, *p* < 0.001 (one-way ANOVA with Tukey’s multiple comparison test). Scale bars; 100 μm **(A)**, 5 μm **(B)**.

We examined the possibility of structural plasticity in synaptic connections from the OB to OT domains. Axons of mCherry(+) OB mitral/tufted cells distributed in the layer Ia (the superficial portion of layer I) (Figure 9A). Axonal boutons in mCherry(+) mitral/tufted cells in the amOT and lOT were identified based on VGLUT1 coexpression (Figure 9B), and axonal bouton size was examined using confocal microscopy.

In control mice, axonal bouton size did not differ significantly between the amOT and lOT (Figure 9C). In mice trained to perform reward-pursuing behavior, axonal boutons became larger in the amOT than in the lOT (*F*_(3, 521)_ = 38.52, *p* < 0.0001; Figure 9C left panel; Supplementary Table S4 for multiple comparisons). In mice trained to perform shock avoidance behavior, axonal boutons became larger in the lOT than in the amOT (*F*_(3, 358)_ = 29.41, *p* < 0.0001; Figure 9C right panel; Supplementary Table S4). The numbers of axonal boutons analyzed were 95, 94, 163 and 173 from left to right columns of Figure 9C for attraction learning, and 93, 97, 85 and 87 for aversion learning, from 4 mice in each experimental condition. These results indicate that the association between OB neuron photostimulation and either reward or shock induced amOT- or lOT-specific axonal bouton size development.

### Apposition of synaptic boutons of OB neurons to postsynaptic structures in OT domains

**Figure 10:**
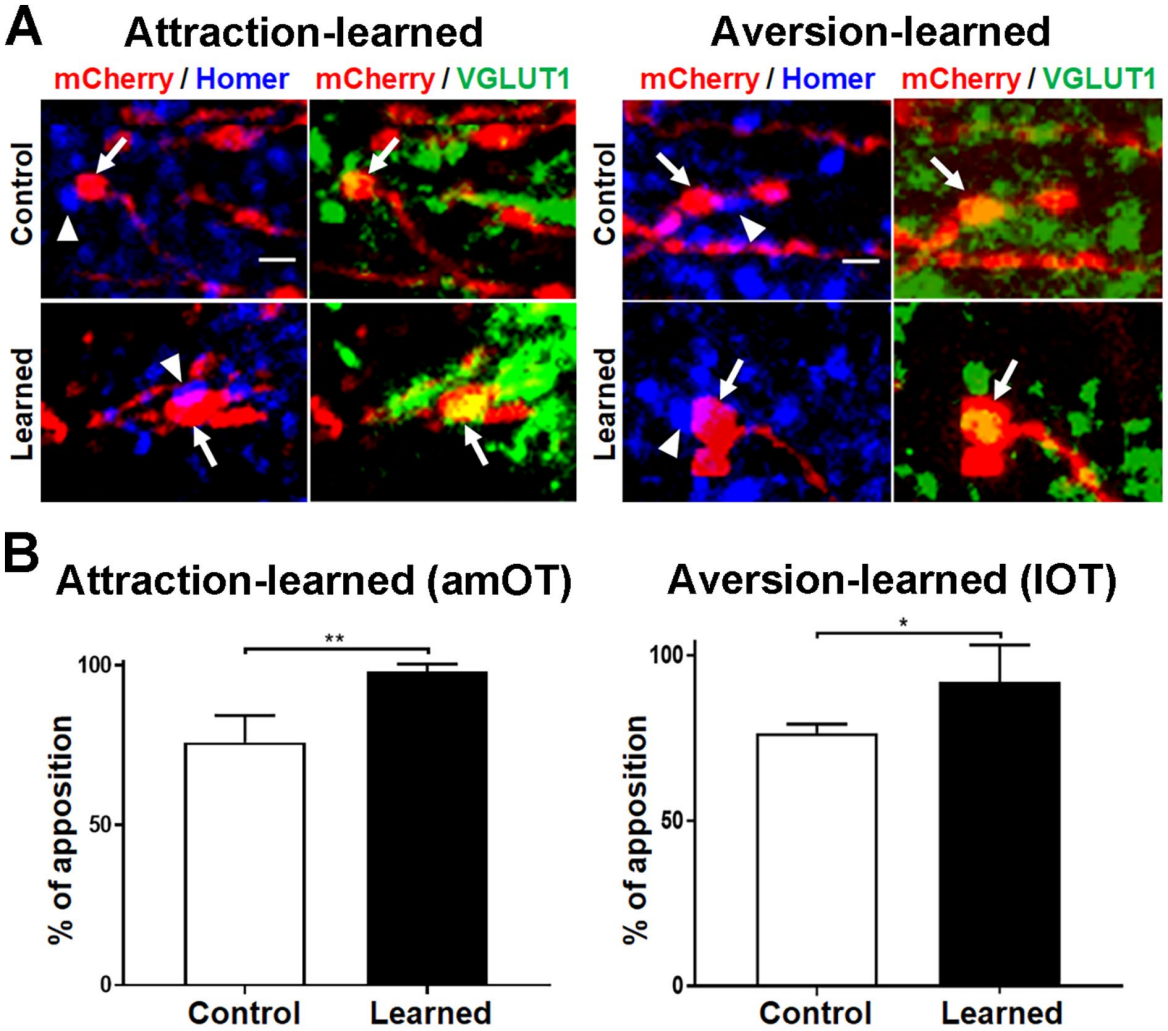
Apposition of OB neuron axonal boutons to postsynaptic molecules. **(A)** Apposition of mCherry (red)/VGLUT1 (green) coexpressing OB neuron axonal boutons (arrows) to Homer 1b/c puncta (blue) (arrowheads). Photographs in amOT layer Ia for the control and attraction-learned mice (left panels) and in lOT layer Ia for the control and aversion-learned mice (right panels) are indicated. Scale bar, 1 μm. **(B)** Percentages of apposition of axonal boutons to Homer 1b/c puncta in the amOT for the control and attraction-learned mice (left panel) as well as in the lOT for the control and aversion-learned mice (right panel). Average ± SD; *n* = 4 in each column, from rostrocaudally distinct 2 areas of layer Ia of 2 mice. *, *p* < 0.05; **, *p* < 0.01 (*t*-test).

To address whether mCherry(+) axonal boutons made synaptic contact with postsynaptic structures, we performed immunohistochemical analysis of Homer 1b/c in the amOT and lOT (Figure 10). Most mCherry(+) and VGLUT1(+) axonal boutons, including large ones in learned mice (Figure 10A lower panels), were apposed to the Homer 1b/c puncta. More than 75 and 90% of the axonal boutons were closely opposed to Homer 1b/c puncta in the amOT of control and attraction-learned mice, respectively (*p* = 0.002; Figure 10B left panel) as well as in the lOT of control and aversion-learned mice, respectively (*p* = 0.035; Figure 10B right panel). The total numbers of axonal boutons analyzed were 38 (control) and 44 (learned) for attraction learning and 47 (control) and 53 (learned) for aversion learning. The results suggest proper synapse formation of mCherry(+) axonal boutons.

## Discussion

### Structural plasticity of axonal boutons projecting to OT domains

In this study, photoactivation of projection neurons expressing ChR2 in the OB and PC was associated with either food reward or foot shock in mice. For both PC to OT and OB to OT connections, axonal boutons in ChR2-expressing neurons preferentially increased in size in the amOT compared to the lOT in mice that had learned reward-pursuing behavior, and increased in the lOT compared to amOT in mice that had learned shock-aversive behavior.

Synaptic structure plasticity is generally correlated with synaptic strength (Lisman and Harris, 1993). For instance, axonal boutons became larger in the strengthened synapses of hippocampal neurons, and their size was correlated with the number of docked vesicles (Murthy et al., 2001). Moreover, alteration of sensory experience strengthened excitatory connections with larger axonal varicosities in whisker barrel cortices (Cheetham et al., 2014), and monocular visual deprivation decreased the size of large axonal boutons and increased that of small boutons in the excitatory synapses of the visual cortex (Sammons et al., 2018). Our findings demonstrated the structural plasticity of excitatory axonal boutons in the OT domains. The learning- and OT domain-specific size development of axonal boutons likely potentiated synaptic strength, which is considered to underlie the activation of specific OT domains in a learning-dependent manner. We confirmed that most ChR2/mCherry and VGLUT1-exprssing axonal boutons were apposed to the Homer1b/c puncta, a scaffold protein of postsynaptic density (de Bartolomeis et al., 2022). Because synaptic plasticity occurs in the coordination of pre- and post-synapses (Lisman and Harris, 1993), postsynaptic plasticity could occur, such as the enlargement of dendritic spines in OT neurons apposing enlarged axonal boutons. Further study of these processes is required.

### ChR2-mCherry-expressing neuronal population and layer distribution of their synaptic connection to OT domains

In the aPC, ChR2-mCherry was expressed in layers IIb and III (Figure 1). aPC neurons in layers IIb and III have been shown to project heavily to other brain regions, whereas aPC neurons in layer IIa project mainly within the aPC (Haberly and Price, 1978). Thus the aPC neurons examined in this study appear to represent major projections from the aPC to the OT. ChR2-mCherry-expressing aPC neurons projected to layers Ib, II, and III in the OT (Figure 4). This layer distribution of intracortical synapses in the OT was similar to that in the aPC, where intracortical synapses terminate in layers Ib, II, and III (Neville and Haberly, 2004).

In the OB, ChR2-mCherry was expressed mainly in mitral/tufted cells of the lateral wall (Figure 6). Mitral cells in the dorsal and ventral OB project to distinct areas of the amygdaloid complex and mediate aversive and attractive behaviors, respectively (Mori and Sakano, 2021). Although the specification of mitral/tufted cells in the lateral wall has not yet been observed, our results demonstrated their structural plasticity to strengthen synaptic connections to distinct OT domains in a learning-dependent manner. As reported previously (Igarashi et al., 2012), we observed heavy axonal projection of mitral/tufted cells to the lOT compared to the amOT, both of which showed structural plasticity. ChR2/mCherry-expressing mitral/tufted cells projected to layer Ia in the OT (Figure 9), indicating a similar distribution to that in the PC (Neville and Haberly, 2004).

This layer distribution of intracortical and sensory synaptic connections is consistent with a previous LFP analysis in the guinea pig OT (Carriero et al., 2009). The present findings indicate that synaptic connectivity in the OT is similar to that in the aPC, as a constituent of three-layered olfactory cortex (Wiegand et al., 2011).

### Plasticity of sensory and intracortical synaptic connections in the OT

Plasticity in cortical networks underlies information storage, learning, and adaptive behavior (Feldman, 2009). Sensory synapses in the PC are thought to be hard-wired compared to plastic intracortical associations (Kanter and Haberly, 1990; Bekkers and Suzuki, 2013). Long-term potentiation in the sensory synapses occurs only during the postnatal period, whereas that in intracortical associational synapses continues through adulthood (Poo and Isaacson, 2007). Spike time-dependent plasticity can be elicited in associational synapses but not in sensory synapses (Johenning et al., 2009). However, some studies have reported plasticity in the sensory synapses. During odor-associated learning, synaptic connections from the OB to the PC were enhanced *in vivo* (Cohen et al., 2015). The activation of sensory synapses at theta frequency induced strong and robust long-term potentiation (Kumar et al., 2021). Thus, the plasticity of sensory synapses in the olfactory cortex remains controversial.

In the present study, both intracortical and sensory synapses to OT domains exhibited structural plasticity in adult mice. Structural plasticity of the intracortical synapses from the aPC to the OT is consistent with their function in influencing OT neuronal activity (White et al., 2019). The structural plasticity of sensory synapses from the OB to the OT domains is intriguing. Among many neuronal routes for sensory signals to reach the OT, the shortest is that from olfactory sensory neurons to mitral/tufted cells and then to OT neurons, which consists of only two synaptic transmission steps. This route appears to be a “highway” for odor information to induce odor-guided behaviors. Learning-dependent plasticity of the sensory connection from the OB to OT domains may represent strong adaptive linkage of odor information to behavioral outputs (Doty, 1986). In drosophila, appetitive odor learning reorganized structural connectivity from the antennal lobe, the homolog of mammalian OB, to the mushroom body, the homolog of the olfactory cortex, in an odor input-specific manner (Baltruschat et al., 2021). In zebrafish, appetitive odor learning induced remapping of odor representation in the posterior zone of the dorsal telencephalon, the homolog of olfactory cortex, while underlying plasticity in neuronal connectivity remains unknown (Frank et al., 2019). Circuit reorganization of the olfactory cortical area by associating odor input with valence would be the common mechanism of odor behavior learning across species, where structural plasticity of sensory synapses may be involved.

The mechanism by which intracortical and sensory synaptic inputs work separately and coordinately in physiological odor learning remains unknown. The OT receives associational synaptic inputs from many brain areas, including the olfactory cortex, amygdala, prefrontal cortex, and ventral tegmental area (Haberly and Price, 1978; Berendse et al., 1992; de Olmos and Heimer, 1999; Zhang et al., 2017b; White et al., 2019; Cansler et al., 2023). These brain regions are mutually connected, and the activation of specific OT domains is likely due to the integration of multiple synaptic inputs involving a variety of brain regions. The present observation of c-fos expression in OT neurons does not necessarily indicate that activation was induced directly via photoactivated synaptic inputs. The combinatory role of multiple synaptic inputs in OT domains in physiological olfactory behavior learning remains to be clarified in future studies.

### Possible mechanisms of structural plasticity in OT synapses and limitations of this study

Dopaminergic signals play a crucial role in OT-mediated odor learning (Zhang et al., 2017a). Dopaminergic signals potentiate synaptic plasticity in OT neurons and reinforce reward prediction coding (Wieland et al., 2015; Oettl et al., 2020). The distinct role of dopaminergic input to the medial and lateral OT in neuronal activity (Bhimani et al., 2022) suggests that OT domain-specific dopaminergic input may be involved in the OT domain-specific structural plasticity of axonal boutons. Other than dopamine, OT receives a variety of neuromodulatory signals (Cansler et al., 2020), and biased neuromodulatory signals among OT domains are suggested (Nogi et al., 2020). Differential OT domain-specific neuromodulatory signals during distinct olfactory behavior learning may induce OT domain-specific synaptic plasticity.

The OT contains D1- and D2-type dopamine receptor-expressing neurons with distinct functions (Murata et al., 2015, 2019; White et al., 2019; Gadziola et al., 2020; Martiros et al., 2022). Our study did not address postsynaptic cell types in contact with axonal boutons. The learning-dependent structural plasticity of axonal boutons may be specific to either D1 or D2 cells.

We used optogenetics to activate specific neurons of the olfactory circuitry. However, optogenetic activation cannot fully reproduce odor-induced physiological activity. We attempted to mimic the temporal properties of odor-induced activity, such as beta range activity in the PC (Figure 2) and beta – low gamma activity in the OB (Figure 7; Martin and Ravel, 2014; Frederick et al., 2016). Of interest, we observed oscillatory activity in the OB associated with continual photostimulation for 500 ms, which likely resulted from intrinsic properties of the neuronal circuitry and may leave room for analysis of its plastic modulation by learning processes. In preliminary experiments we also applied continual photostimulation to PC but failed to generate effective unit or oscillatory activity, which may have been due to differences in the intrinsic circuit properties of the OB and PC. A temporal property that could not be mimicked was a link with respiration cycles occurring in the odor-induced activity (Mori and Sakano, 2022). The connection and influence of respiratory rhythm generator neurons to adrenergic neurons and on behavioral arousal (Yackle et al., 2017) raise the possibility that respiration-related brain state changes may play important roles in information processing and neuronal plasticity. Such potential deficiencies of optogenetics must be taken into consideration especially for its application to the olfactory system. Despite these limitations, our findings revealed the plastic potential of sensory and intracortical synapses in the OT, which is thought to contribute to the highly adaptive property of odor-guided motivated behaviors.

## Data availability statement

The original contributions presented in the study are included in the article/Supplementary material, further inquiries can be directed to the corresponding author.

## Ethics statement

The animal study was approved by Kochi Medical School Animal Care and Use Committee. The study was conducted in accordance with the local legislation and institutional requirements.

## Author contributions

MS, YK, and MY contributed to initial conception, design of the study, organized and conducted experiments. YM and MT helped improve the conception and study design throughout the study. MS and MY wrote the manuscript. All authors contributed to the article and approved the submitted version.

## Funding

This study was supported by the Grant-in-Aid for Scientific Research from Japan Society for Promotion of Science (grant nos. 19H03341 and 22H02734) and the research grant from the Uehara Memorial Foundation.

## Conflict of interest

The authors declare that the research was conducted in the absence of any commercial or financial relationships that could be construed as a potential conflict of interest.

## Publisher’s note

All claims expressed in this article are solely those of the authors and do not necessarily represent those of their affiliated organizations, or those of the publisher, the editors and the reviewers. Any product that may be evaluated in this article, or claim that may be made by its manufacturer, is not guaranteed or endorsed by the publisher.
