# Supplementary material for: Learning-dependent structural plasticity of intracortical and sensory connections to functional domains of the olfactory tubercle
Source: Front Neurosci. 2023 Aug 23;17:1247375. doi: 10.3389/fnins.2023.1247375 (PMC10480507; doi:10.3389/fnins.2023.1247375)
Supplement: Supplementary file 1 [file Data_Sheet_1.docx]

Supplementary Material

Learning-dependent structural plasticity of intracortical and sensory connections to functional domains of the olfactory tubercle

Md Fazley Rabbi Sha, Yuriko Koga, Yoshihiro Murata, Mutsuo Taniguchi, Masahiro Yamaguchi*

Department of Physiology, Kochi Medical School, Kochi University, Kochi, Japan

*** Correspondence:**

Masahiro Yamaguchi
[yamaguchi@kochi-u.ac.jp](mailto:yamaguchi@kochi-u.ac.jp)


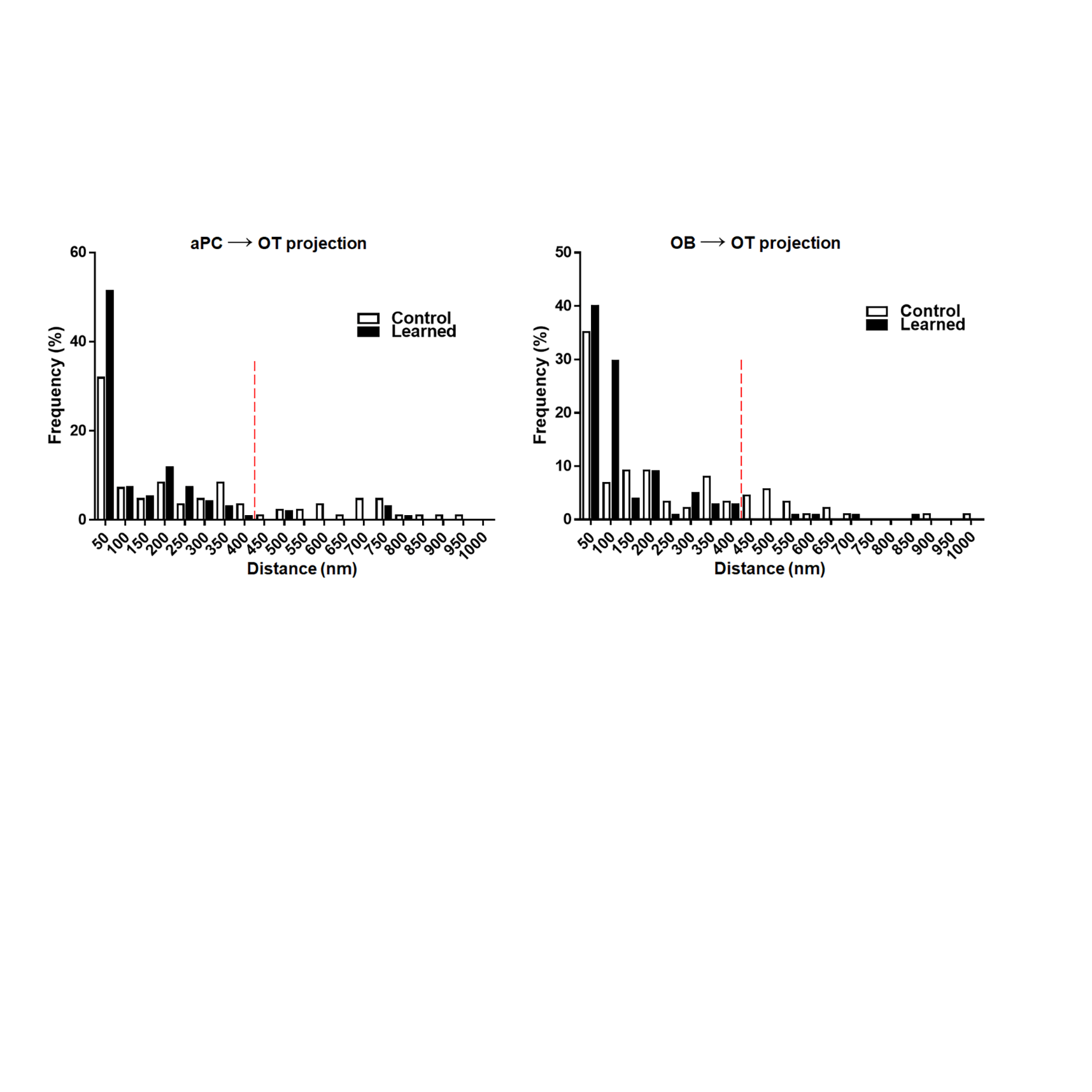


**Supplementary Fig. 1. Distance between mCherry(+) axonal boutons and Homer 1b/c puncta**

Distances between mCherry(+) axonal boutons and the nearest Homer 1b/c puncta in X-Y planes of confocal images were measured, and the distribution of the distances was expressed as the frequency (%) among all the measurements. (Left panel) Measurements of axonal boutons of aPC neurons in the amOT layers Ib, II and III for control and attraction-learned mice and in the lOT layers Ib, II and III for control and aversion-learned mice. (Right panel) Measurements of axonal boutons of OB mitral/tufted cells in the amOT layers Ia for control and attraction-learned mice and in the lOT layer Ia for control and aversion-learned mice. Red dotted lines (at 400 nm) indicate the threshold for the judgement of close apposition. The numbers of boutons analyzed were 81 (control) and 91 (learned) for aPC neurons, and 85 (control) and 97 (learned) for OB mitral/tufted cells.

**Supplementary Table 1. Statistical results of Tukey's multiple comparison test for Fig. 3**

Comparisons of c-fos(+) cell density in the photostimulation of aPC neurons. Excerpts from all compared pairs are indicated.

**Supplementary Table 2. Statistical results of Tukey's multiple comparison test for Fig. 4**

Comparisons of axonal bouton size in the photostimulation of aPC neurons. Excerpts from all compared pairs are indicated.

**Supplementary Table 3. Statistical results of Tukey's multiple comparison test for Fig. 8**

Comparisons of c-fos(+) cell density in the photostimulation of OB neurons. Excerpts from all compared pairs are indicated.

**Supplementary Table 4. Statistical results of Tukey's multiple comparison test for Fig. 9**

Comparisons of axonal bouton size in the photostimulation of OB neurons. Excerpts from all compared pairs are indicated.
